# The genetics of phenotypic plasticity in nematode feeding structures

**DOI:** 10.1098/rsob.160332

**Published:** 2017-03-15

**Authors:** Ralf J. Sommer, Mohannad Dardiry, Masa Lenuzzi, Suryesh Namdeo, Tess Renahan, Bogdan Sieriebriennikov, Michael S. Werner

**Affiliations:** Department for Integrative Evolutionary Biology, Max-Planck Institute for Developmental Biology, Spemannstrasse 37, 72076 Tübingen, Germany

**Keywords:** phenotypic plasticity, *Pristionchus pacificus*, switch genes, nuclear hormone receptors, epigenetics

## Abstract

Phenotypic plasticity has been proposed as an ecological and evolutionary concept. Ecologically, it can help study how genes and the environment interact to produce robust phenotypes. Evolutionarily, as a facilitator it might contribute to phenotypic novelty and diversification. However, the discussion of phenotypic plasticity remains contentious in parts due to the absence of model systems and rigorous genetic studies. Here, we summarize recent work on the nematode *Pristionchus pacificus,* which exhibits a feeding plasticity allowing predatory or bacteriovorous feeding. We show feeding plasticity to be controlled by developmental switch genes that are themselves under epigenetic control. Phylogenetic and comparative studies support phenotypic plasticity and its role as a facilitator of morphological novelty and diversity.

## 1. Introduction

All organisms have to adapt to the environment and to environmental variation. Often, alternative conditions result in different expressions and values of traits, a phenomenon referred to as ‘phenotypic plasticity’. Generally, phenotypic (or developmental) plasticity is defined as the property of a given genotype to produce different phenotypes depending on distinct environmental conditions [1,2]. In addition to being an ecological concept that allows studying how organisms respond to environmental variation, phenotypic plasticity also represents an integral part of the evolutionary process. Given these ecological and evolutionary implications, it is not surprising that the concept of phenotypic plasticity has been contentious ever since its introduction at the beginning of the 20th century. For some, plasticity is the major driver and facilitator of phenotypic diversification, and, as such, of greatest importance for understanding evolution and its underlying mechanisms [1–3]. For others, phenotypic plasticity represents environmental noise and is sometimes considered to even hinder evolution because environmentally induced variation may slow down the rate of adaptive processes [4,5]. This controversy largely depends on two limitations. First, there is confusion over the different types of plasticity found in nature. Plasticity can be adaptive or non-adaptive, reversible or irreversible, conditional or stochastic, and continuous or discrete, all of which require careful evaluations of examples of plasticity for their potential evolutionary significance. Second, the absence of plasticity model systems has long hampered the elucidation of potential molecular and genetic mechanisms, the identification of which would provide a framework for theoretical considerations.

In 1965, Bradshaw made one of the most important contributions to the concept of phenotypic plasticity when he proposed that plasticity must have a genetic basis. This idea grew out of the observation that the plasticity of a trait is independent of the phenotype of the plastic trait itself [6]. However, little progress was made to identify underlying mechanisms, largely due to the absence of laboratory model systems of plasticity. Here, we summarize recent studies on phenotypic plasticity of feeding structures in the nematode *Pristionchus pacificus*. The advantages of this system have allowed unbiased genetic approaches that provide detailed insight into the genetic control of plasticity and a molecular framework for studying the mechanisms of plasticity and genetic–environmental interactions. A model system approach in nematodes might therefore help clarify the role of plasticity in evolution by shedding light on its molecular mechanisms and macro-evolutionary potentials. We will start with a brief historical account of phenotypic plasticity and its role for the evolution of novelty.

## 2. A historical account

The history of phenotypic plasticity begins at the beginning of the 20th century (table 1) [7]. In 1909, Richard Woltereck carried out the first experiments on plastic characters using the water flea *Daphnia*. He coined the term ‘reaction norm’ (or norm of reaction) to describe the relationship between the expressions of phenotypes across a range of different environments [3]. However, it was Johannsen (1911) who first distinguished between genotype and phenotype, and thereby introduced the concept of genotype–environment interaction [8]. This concept was only developed further three decades later by the Russian biologist Schmalhausen and the British developmental biologist Waddington. In particular, Waddington, using environmental perturbation of development, provided important conceptual contributions [9]. For example, he introduced the concept of genetic assimilation based on his work with the bithorax and crossveinless phenotypes in *Drosophila*. When fly pupae were exposed to heat shock, some of them developed a crossveinless phenotype. Upon artificial selection for multiple generations, this trait became fixed in some animals even without heat shock. Similarly, when flies were treated with ether vapour, some exhibited a homeotic bithorax phenotype, which again could be fixed even without ether induction after artificial selection for approximately 20 generations. Waddington argued that genetic assimilation allows the environmental response of an organism to be incorporated into the developmental programme of the organism. While it is now known that the fixation of the bithorax phenotype was based on the selection of standing genetic variation at a homeotic gene [10], at the time these findings were controversially discussed and often referred to as Lamarckian mechanisms. Given the missing genetic foundation of development and plasticity in the 1940s, it is not surprising that Waddington's claim for an extended evolutionary synthesis found little support among neo-Darwinists [11].

**Table 1.** History of phenotypic plasticity.

Date	Scientist(s)	Theory
1909	Woltereck	reaction norm
1913	Johannsen	genotype–phenotype distinction
1940–1950	Waddington Schmalhausen	canalization/assimilation
1965	Bradshaw	genetic basis of plasticity
1998–2003	Schlichting/Pigliucci West-Eberhard	facilitator hypothesis

The major conceptual advancement for plasticity research was in 1965 when Anthony Bradshaw proposed that phenotypic plasticity and the ability to express alternative phenotypes must be genetically controlled [6]. Some plants develop alternative phenotypes in response to extreme environmental conditions. Using a comparative approach, Bradshaw realized that the plasticity of a trait could differ between close relatives of the same genus, independent of the trait itself. From this observation he concluded that the genetic control of a character is independent of the character's plasticity. This remarkable conclusion represents one of the most important testimonies of the power of comparative approaches and the key foundation for modern studies of plasticity.

It is not surprising that botanists have paid detailed attention to reaction norm and plasticity for breeding purposes, and the first modern monographs that advertised the significance of phenotypic plasticity for development and evolution were written by active practitioners in this field [3]. Many examples of plasticity from animals are known as well, often in insects. The migratory locust *Schistocerca gregaria* can form two alternative phenotypes in relation to food availability. Adult *Schistocerca* are dark with large wings when food is abundant, whereas they are green with small wings when food is limited [12]. Similarly, many butterflies are known to form distinct wing patterns in the dry and rainy season in the tropics or in spring and summer in more temperate climates [13]. Perhaps the most spectacular examples of plasticity are those found in hymenopterans forming the basis for eusociality in insects and resulting in the most extreme forms of morphological and behavioural novelties. Mary-Jane West-Eberhard, after a long and active career studying social behaviours in Hymenoptera, proposed an extended evolutionary theory that links development and plasticity to evolution. Her monograph *Developmental plasticity and evolution* provides an exhaustive overview on alternative phenotypes in nature [2]. Building on the now available genetic understanding of developmental processes, she proposed plasticity to represent a major facilitator and driver for the evolution of novelty and the morphological and behavioural diversification in animals and plants.

This long path from Johannsen, Waddington and Bradshaw to current plasticity research has resulted in a strong conceptual framework for the potential significance of plasticity for evolution (table 1). However, scepticism remains, largely due to the near absence of associated genetic and molecular mechanisms of plasticity [14]. To overcome these limitations, plasticity research requires model systems that tie developmental plasticity in response to environmental perturbations to laboratory approaches. Before summarizing the recent inroads obtained in one laboratory model for phenotypic plasticity, the next paragraph will briefly summarize the different forms of plasticity.

## 3. Some important terminology: the different forms of plasticity

By definition, the concept of phenotypic plasticity incorporates many unrelated phenomena, which has resulted in enormous confusion and debate about its potential for evolutionary adaptations [15]. Three major distinctions are necessary to properly evaluate the potential significance of plasticity for evolution. First, phenotypic plasticity can be adaptive or non-adaptive, and only the former can contribute to adaptive evolution when organisms are faced with a new or altered environment. In contrast, non-adaptive plasticity in response to extreme and often stressful environments is likely to result in maladaptive traits that are without evolutionary significance [15].

Second, plasticity can be continuous or discrete, the latter resulting in alternative phenotypes often referred to as polyphenisms. Such alternative phenotypes have several advantages for experimental analysis and evaluation in the field. Most importantly, alternative phenotypes can more readily be distinguished from genetic polymorphisms that can also result in phenotypic divergence. Multiple examples of polyphenisms from aerial and subterranean stem and leaf formation in water plants, insect wing and body form dimorphisms and the casts of social insects have been studied in detail to analyse the interaction between the genotype and the environment in the specification of plastic traits [2]. The binary readout of alternative phenotypes provides a major advantage of such experimental analyses.

Third, plasticity might be regulated by conditional and stochastic factors [16]. While the former is more common, additional stochastic elements of regulation are known in some examples of plasticity and such cases have several experimental advantages. Most examples of plasticity have environment *a* inducing phenotype **A** and environment *b* inducing phenotype **B**. However, organisms might form alternative phenotypes **A** and **B** in part due to stochastic factors that are independent of environmental alterations. The potential role of stochastic factors has been largely overlooked in plant and animal systems, but is well known in microbes. Phenotypic heterogeneity or bistability is known in many bacteria to result in phenotypically distinct subpopulations of cells [17,18]. Persister cell formation in *Staphylococcus aureus* and spore formation in *Bacillus subtilis* represent just a few examples of phenotypic heterogeneity that occur to a certain extent in a stochastic manner. Antibiotic resistance seen by persister cells resulted in detailed molecular and mechanistic insight into the stochastic regulation of phenotypic heterogeneity [19].

Adaptive versus non-adaptive, continuous versus discrete, and conditional versus stochastic regulation of plasticity represent important distinctions for the evaluation and significance of plastic traits in development and evolution. However, one additional factor that often complicates a proper evaluation of plasticity is the inherent difficulty to distinguish between genetic polymorphisms and polyphenisms. Genetic polymorphisms are a cornerstone of mainstream evolutionary theory for the generation of phenotypic divergence. Therefore, empirical studies on plasticity would profit from a proper distinction between polymorphisms and polyphenisms. Besides inbred lines in outbreeding species, self-fertilization in hermaphroditic organisms results in isogenic lines. Such isogenic lines can rule out contributions of genetic polymorphisms. Some plants, nematodes and other animals with a hermaphroditic mode of reproduction are therefore ideal for studies of plasticity, mimicking the isogenic advantages of bacteria with phenotypic heterogeneity.

In the following, we summarize recent insight into the genetic regulation of a mouth-form feeding plasticity in the nematode *P. pacificus*. This example of plasticity is adaptive, represents a dimorphic trait with two alternative phenotypes, and contains conditional and stochastic elements of regulation. *Pristionchus pacificus* is a hermaphroditic species with isogenic propagation, and is amenable to forward and reverse genetic analysis [20,21]. We begin with a brief summary of mouth-form polyphenism in this nematode species.

## 4. Mouth-form polyphenism as a case study

**Figure 1.**
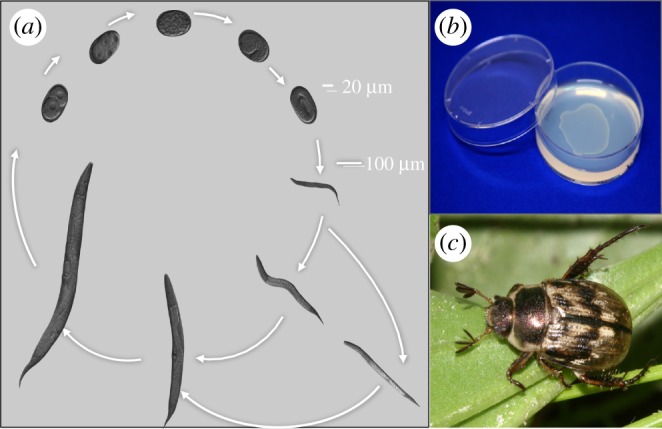
*Pristionchus pacificus* and growth. (*a*) Adult hermaphrodites lay eggs that develop through four larval stages to become adult. The first juvenile stage remains in the eggshell in *P. pacificus*. Under harsh and unfavourable conditions, worms develop into an arrested and long-lived dauer stage. (*b*) In the laboratory, worms are grown on agar plates with *Escherichia coli* as food source. Under these conditions, worms complete their direct life cycle in 4 days (20°). (*c*) The oriental beetle *Exomala* *orientalis* from Japan and the United States is one of the scarab beetle hosts on which *P. pacificus* is found in the dauer larval stage.

The genus *Pristionchus* belongs to the nematode family Diplogastridae, which shows entomophilic associations (figure 1) and omnivorous feeding strategies, including predation on other nematodes [22]. Usually, nematodes stay in the arrested dauer stage—a nematode-specific form of dormancy—in or on the insect vector (figure 1*a*). Nematode–insect associations represent a continuum between two most extreme forms, with dauer larvae of some species jumping on and off their carriers (phoresy), whereas others wait for the insect to die in order to resume development on the insect carcass (necromeny). Insect carcasses represent heterogeneous environments full of a variety of microbes. Such insect carcasses are best characterized by a boom and bust strategy of many of its inhabitants. While many nematodes, yeasts, protists and bacteria are known to proliferate on insect cadavers, few, if any, of these systems have been fully characterized, in particular with regard to species succession during decomposition.

**Figure 2.**
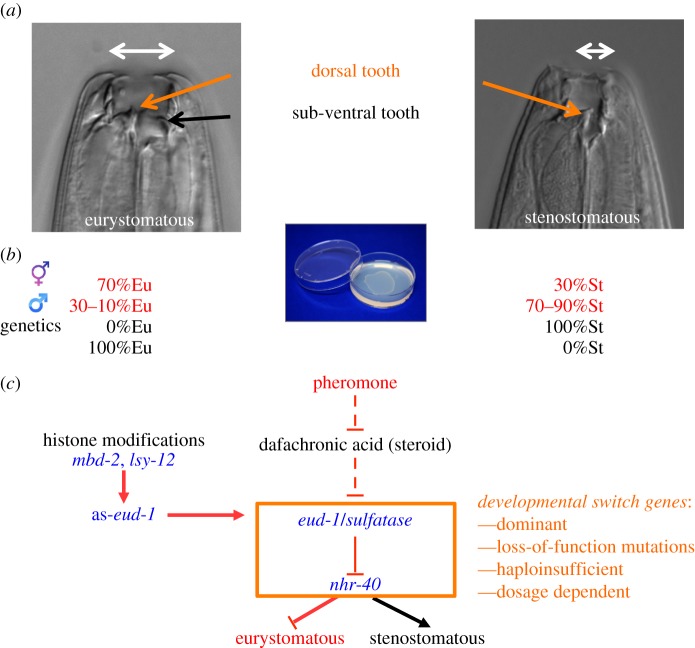
Genetic regulation of phenotypic plasticity of *P. pacificus* feeding structures. (*a*) Mouth dimorphism. During larval development, *P. pacificus* individuals make an irreversible decision to develop a eurystomatous morph with two teeth (orange and black arrows) and a broad buccal cavity (white arrow), or alternatively, a stenostomatous morph with a single dorsal tooth (orange arrow) and a narrow buccal cavity (white arrow). (*b*) Under fixed laboratory conditions, mouth-form plasticity shows stochastic regulation resulting in hermaphrodites having approximately 70% eurystomatous mouth-forms, whereas males have been 10–30% eurystomatous animals. In genetic screens, monomorphic mutants can be isolated that are either 100% stenostomatous or 100% eurystomatous. (*c*) Partial genetic network regulating mouth-form plasticity. The sulfatase-encoding *eud-1* gene and the nuclear hormone receptor are developmental switch mutations, which are dominant, *loss-of-function* and dosage dependent, resulting in all-stenostomatous or all-eurystomatous phenotypes, respectively. Small molecule signalling acts upstream of *eud-1* and involves pheromones and steroid hormone signalling, which are not a subject of this review. Histone modifications are crucial for mouth-form regulation and act through an antisense message at the *eud-1* locus (as-*eud-1*).

*Pristionchus pacificus* and related nematodes live preferentially on scarab beetles (i.e. cockchafers, dung beetles and stag beetles; figure 1*c*) [23]. On living beetles, *P. pacificus* is found exclusively in the arrested dauer stage and decomposition experiments indicate that adult worms are found on the cadaver only 7 days after the beetle's death [24]. *Pristionchus* and other nematodes live on and wait for the beetle to die, resulting in enormous competition for food and survival on the carcass. It was long known that *Pristionchus* and other diplogastrid nematodes form teeth-like denticles in their mouths, which allow predatory feeding (figure 2*a*) [25]. Also, it was long known that many species form two alternative mouth-forms. In the case of *P. pacificus*, animals decide during larval development in an irreversible manner to adopt a eurystomatous (Eu) or a stenostomatous (St) mouth-form (figure 2*a*) [25]. Eu animals form two teeth with a wide buccal cavity, representing predators. In contrast, St animals have a single tooth with a narrow buccal cavity and are strict microbial feeders. Selection experiments have shown that the mouth-form dimorphism represents an example of phenotypic plasticity because isogenic animals can form both mouth-forms [25]. The dimorphism is discrete and adaptive with strong fitness effects preferring St and Eu animals under bacterial and predatory conditions, respectively [26,27]. Most importantly, mouth-form plasticity is regulated by conditional factors such as starvation and crowding [25], but also contains stochastic elements of regulation. Specifically, a nearly constant ratio of 70–90% Eu : 30–10% St animals is formed under fixed environmental conditions (figure 2*b*). It is this aspect of stochastic regulation resulting in the occurrence of both mouth-forms under standard laboratory conditions that allows manipulation of plasticity by genetic, molecular and chemical tools [16].

## 5. Genetics of nematode feeding plasticity

*Pristionchus pacificus* has been developed as a model system in evolutionary biology [20,21]. While only distantly related to *Caenorhabditis elegans*, it shares a number of features: self-fertilization, a short generation time of 4 days and monoxenic growth on *E. coli*. Adopting the functional toolkit of *C. elegans*, forward and reverse genetic tools are available in *P. pacificus*, including CRISPR-Cas9 genetic engineering and genetic transformation [28,29]. In addition, the known beetle association allowed a vast collection of *P. pacificus* strains and genomes to be catalogued [30,31].

Given the stochastic mouth-form dimorphism of wild-type *P. pacificus* animals when grown on bacteria, mutagenesis screens for monomorphic mutants can be performed to isolate strains deficient in the formation of one particular mouth-form (figure 2*b*). The first such unbiased genetic screen resulted in a *eu*rystomatous-form *d*efective mutant, *eud-1*, which turned out to be dominant and represents a developmental switch gene (figure 2*c*) [32]. Mutant *eud-1* animals are all-St, resulting in the complete absence of Eu animals. In contrast, overexpression of *eud-1* in wild-type or *eud-1* mutant animals reverts this phenotype to all-Eu. These and other experiments showed that *eud-1* is haplo-insufficient and dosage dependent. *eud-1* alleles are dominant, and their all-St phenotype results from reduction-of-function, but not gain-of-function mutations. Consistently, *eud-1* mutant alleles were rescued with a wild-type copy of *eud-1*, whereas overexpression of a mutant copy of the gene did not result in any phenotype, as would usually be the case for gain-of-function mutations (figure 2*c*) [32].

A suppressor screen for Eu animals in an all-St *eud-1* mutant background resulted in the identification of the nuclear hormone receptor *nhr-40* (figure 2*c*) [33]. Interestingly, *nhr-40* is also part of the developmental switch constituting similar genetic features but with an opposite phenotype to *eud-1*: *nhr-40* mutants are all-Eu, while overexpression results in all-St lines. *nhr-40* mutants are again dominant as loss-of-function mutants and haplo-insufficient. Thus, two genes regulating mouth-form plasticity show a dominant null or reduction-of-function phenotype. This is in strong contrast to the overall pattern in nematodes. Screens for dominant mutations in *C. elegans* resulted in many gain-of-function alleles, whereas *unc-108* represents the only gene that when mutated results in a dominant null phenotype, indicating haplo-insufficient genes to be rare [34].

Together, the experiments summarized above allow four major conclusions. First, unbiased genetic analysis of *P. pacificus* feeding plasticity indicates that plasticity is indeed under genetic control. *eud-1* and *nhr-40* mutants are monomorphic, being either all-St or all-Eu. Thus, genes affect mouth-form plasticity without affecting the character state itself; in *eud-1* mutants the St mouth-form is properly formed, similar to the Eu form in *nhr-40* mutant animals. Second, both genes are part of a developmental switch with loss-of-function and overexpression, resulting in complete but opposite phenotypes. Developmental switches had long been predicted to play an important role in plasticity regulation [2], but due to the previous absence of genetic models of plasticity, little genetic evidence was obtained. Third, *eud-1* and *nhr-40* are both located on the X chromosome. *Pristionchus pacificus* has an XO karyotype in males, similar to *C. elegans* [35]. Interestingly, males have predominantly a St mouth-form [25] and *eud-1* and *nhr-40* mutant males are all-St and all-Eu, respectively. Thus, *eud-1* and *nhr-40* escape male dosage compensation, a process that is just beginning to be investigated in *P. pacificus* [36]. Finally, it is interesting to note that *eud-1* resulted from a recent duplication [32]. While *C. elegans* contains one *eud-1*/sulfatase copy located on an autosome, *P. pacificus* contains three copies, with the two recently evolved genes being located on the X chromosome. However, CRISPR/Cas9-induced mutations in the two other *eud-1*-like genes in *P. pacificus* suggest that there are no specific phenotypes associated with the knockout of both genes [37].

## 6. Epigenetic control of switch genes

Two common aspects of *eud-1* and *nhr-40* mutants resulting in monomorphic, plasticity-defective phenotypes are that they show no other obvious phenotypes. In contrast, an unbiased search for mouth-form defects in a collection of mutants previously isolated for their egg-laying- or vulva-defective phenotypes identified *mbd-2* and *lsy-12* mutants to resemble an all-St *eud-1*-like phenotype [38]. *mbd-2* is egg-laying-defective and encodes a member of the methyl-binding protein family that is strongly reduced in *C. elegans* but not in *P. pacificus* [39,40]. *lsy-12* encodes a conserved histone-acetyltransferase, and *mbd-2* and *lsy-12* mutants were shown to result in massive histone modification defects involving multiple gene activation marks, such as H3K4me3, H3K9ac and H3K27ac [38]. Given that *mbd-2*, *lsy-12* and *eud-1* mutants have nearly identical mouth-form monomorphism, *eud-1* was itself a potential target for histone modification, and indeed *eud-1* expression is downregulated in *mbd-2* and *lsy-12* mutants. Interestingly, however, histone modification defects affect an antisense message at the *eud-1* locus, and overexpression experiments with this as-*eud-1* transcript suggest that as-*eud-1* positively regulates *eud-1* expression [38]. Together, these findings strongly suggest that the developmental switch is under epigenetic control. In principle, the epigenetic regulation of a switch mechanism is ideally suited to incorporate environmental information and environmental variation. However, information about associated mechanisms in *P. pacificus* awaits future studies, whereas several studies in insects recently already indicated the involvement of epigenetic mechanisms in gene-environmental interactions [41–43]. In conclusion, the use of forward genetic approaches in a laboratory model system provide strong evidence for the regulation of nematode feeding plasticity by developmental switch genes. Furthermore, epigenetic mechanisms including histone modifications and antisense RNA-mediated regulation might be crucial for gene–environment interactions.

## 7. Macro-evolutionary potentials

The genetic and epigenetic control of feeding plasticity in *P. pacificus* provides a basis to study how organisms sense and respond to the environment and to environmental variation. But is plasticity also important for evolution? Answering this question requires comparative studies that when performed in a phylogenetic context might provide insight into the significance of plasticity for evolutionary processes. Micro-evolutionary studies, by comparing many different wild isolates of *P. pacificus*, indicated strong differences in Eu : St ratios between isolates that correlated with *eud-1* expression [32]. Two recent studies have moved this analysis to the macro-evolutionary level, suggesting that phenotypic plasticity indeed facilitates rapid diversification. Susoy and co-workers studied the evolution of feeding structures in more than 90 nematode species using geometric morphometrics [44]. These species included dimorphic taxa, such as *P. pacificus*, but also monomorphic species that never evolved feeding plasticity, such as *C. elegans* (primary monomorphic), and those that had secondarily lost it (secondary monomorphic). This study found that feeding dimorphism was indeed associated with a strong increase in complexity of mouth-form structures [44]. At the same time, the subsequent assimilation of a single mouth-form phenotype (secondary monomorphism) coincided with a decrease in morphological complexity, but an increase in evolutionary rates. Thus, the gain and loss of feeding plasticity have led to increased diversity in these nematodes [8].

A second case of mouth-form plasticity increasing morphological diversification came from a striking example of fig-associated *Pristionchus* nematodes. Besides the worldwide branch of the genus that is associated with scarab beetles (currently more than 30 species), a recent study identified *Pristionchus* species, such as *P. borbonicus*, that live in association with fig wasps and figs [16]. These nematodes are extraordinarily diverse in their mouth morphology for two reasons. First, *P. borbonicus* and others form five distinct mouth-forms that occur in succession in developing fig synconia, thereby increasing the polyphenism from two to five distinct morphs. Second, the morphological diversity of these five morphs exceeds that of several higher taxa, although all five morphs are formed by the same species [16]. These findings strongly support the facilitator hypothesis, and they also indicate that ecological diversity can be maintained in the absence of genetic variation as all this diversity is seen within a single species and without associated speciation and radiation events [45].

## 8. Perspective

Phenotypic plasticity represents a striking phenomenon observed in organisms of all domains of life. It has been a contentious concept and was partially dismissed by mainstream evolutionary theory because many unrelated phenomena have been inappropriately mixed under the same heading. Following and extending previous attempts by Ghalambor *et al.* [15], we have tried to clarify terminology to provide necessary distinctions that will help study and evaluate plasticity, and establish its significance for evolution. Second, the use of a laboratory model system approach has provided strong evidence for the genetic control of feeding plasticity in *P. pacificus*. This genetic framework can serve as a paradigm to study in detail how the same genotype interacts with the environment to control this plastic trait. Besides nematodes, insects and diverse plants are very important multicellular organisms for the study of phenotypic plasticity. In particular, work on butterfly wing patterns and the coloration of caterpillars, but also horn size in different beetles, provide powerful inroads in the proper evaluation of plasticity [46,47]. Together, these studies on plants, insects and nematodes will provide mechanistic insight into this fascinating biological principle and will help provide an extended framework for evolution.
